# The Utilization of Recycled Masonry Aggregate and Recycled EPS for Concrete Blocks for Mortarless Masonry

**DOI:** 10.3390/ma12121923

**Published:** 2019-06-14

**Authors:** Tereza Pavlu, Kristina Fortova, Jakub Divis, Petr Hajek

**Affiliations:** University Centre for Energy Efficient Buildings of Technical University in Prague, Trinecka 1024, 273 43 Bustehrad, Czech Republic; kristina.fortova@cvut.cz (K.F.); jakub.divis@cvut.cz (J.D.); petr.hajek@fsv.cvut.cz (P.H.)

**Keywords:** recycled masonry aggregate, recycled expanded polystyrene, recycled aggregate concrete, concrete blocks for mortarless masonry, thermal properties, environmentally-based optimization

## Abstract

The main aim of this paper is to carry out the environmentally based enhancement of a concrete mixture containing recycled materials whilst considering natural resource consumption as well as mechanical and thermal property levels. The developed concrete is intended to be used in mortarless masonry wall structures. Ten concrete mixtures with different types and replacement rates of recycled masonry aggregate and recycled expanded polystyrene were prepared, and their mechanical and thermal properties were experimentally investigated. It was found that the use of recycled masonry aggregate led to better thermal properties while maintaining sufficient mechanical properties. On the contrary, the addition of recycled expanded polystyrene did not significantly affect the thermal properties of concrete, but the mechanical properties considerably declined. For this reason, the recycled masonry aggregate is suitable to use as an aggregate for concrete masonry blocks for wall structures.

## 1. Introduction

The effective use of construction and demolition waste (CDW) and its application in reusable structural elements can simultaneously reduce waste dumping and decrease the need to use primary resources, both of which are important environmental aspects to be considered in responsible sustainability management. This represents an important contribution to the solution of one of the core objectives of the 2030 UN Agenda on Sustainable Development—Goal 12: Ensure sustainable consumption and production patterns [1]. This goal is focused on economic growth based on efficient resource use and low environmental degradation while improving the well-being of people. This can be done by a shift towards more sustainable resource consumption and improved production processes.

Currently, this is a very real problem, not only from a local/regional perspective, but especially from a global point of view. The material footprint per capita of developing countries almost doubled in the last eight years, representing a significant and needed improvement in material standards of living [1]. Most of this increase is connected with the rising consumption of nonmetallic minerals due to growth in infrastructure and construction. This also includes the environmental impact of concrete structures, which is still growing. Thus, concrete plays an important role in this process and represents a promising challenge for the future. 

The replacement of natural aggregate (NA) by recycled aggregate (RA) from construction and demolition waste reduces consumption of primary recourses. However, its utilization mostly negatively affects the properties of concrete. For this reason, the utilization of recycled construction and demolition waste is mostly limited to the use of recycled concrete waste for constructing base layers in road structures or partial replacement of aggregates for concrete. Mixed recycled aggregate (MRA) with a high content of waste masonry has not found satisfactory utilization yet. There are concerns over the quality of recycled masonry as a technological material due to the complexity of the demolition and recycling process. Furthermore, this secondary raw material cannot be used as an aggregate for concrete according to Czech standards [2]. However, the selective demolition and two-phase recycling process leads to higher-quality recycled masonry aggregate (RMA) without the unwanted impurities which negatively influence its properties. Despite the high quality of RMA, its use as an aggregate for recycled aggregate concrete (RAC) negatively influences mechanical properties and durability. However, RMA has a positive impact on thermal properties.

Many studies of the properties of RMA and its effect on the mechanical properties of RAC have been published [3,4,5,6,7,8,9,10,11,12,13,14,15,16,17,18,19,20,21,22,23,24,25,26,27,28,29]. It has been recognized that RMA differs from NA mainly in terms of water absorption, density, and resistances, for instance, resistance to wear, abrasion resistance, or freeze–thaw resistance [24]. The range of water absorption of coarse RMA has been established from 10% to 19%, which is up to 25 times higher than natural gravel [5,13,22,25], and fine RMA from 12% to 15%, which is more than 10 times higher than natural sand [16,22,30]. The dry density of coarse RMA ranges between 1800 and 2700 kg/m^3^ and fine RMA between 2000 and 2500 kg/m^3^, which is generally lower than natural gravel and sand [5,13,16,22,25,26].

The physical, mechanical, thermal, and durability properties of concrete are influenced by the physical, mechanical, thermal, and durability properties of aggregates. The higher water absorption of RMA affects the workability of fresh concrete [30]. For this reason, it is necessary to determine the additional water needed and to add it to the concrete mixture during mixing [16] or to soak RMA in water for 24 h before mixing [22] to achieve workability that is similar to a conventional concrete mixture. Workability and the effective water/cement (w/c) ratio influence the compressive strength of concrete [26]. The effective w/c ratio is the total amount of water which reacts with cement divided by the total amount of cement. For recycled aggregate concrete, this depends on the water absorption capacity of the recycled aggregate [31]. The compressive strength of recycled aggregate concrete decreases with an increasing NA replacement rate [16,22,30]. No significant decline of compressive strength has been found for recycled aggregate concrete with 15% coarse RMA. On the contrary, the compressive strength of concrete with full replacement of coarse NA by RMA decreases up to 35% [22]. The partial replacement of natural sand by fine RMA has had no significant impact on the compressive strength of recycled aggregate concrete. The reason could be the silica and alumina contents in crushed bricks, which could lead to pozzolanic reactions [30].

The thermal conductivity of concrete depends on the type of aggregate [32], its thermal conductivity, as well as the density and porosity of the aggregate [33]. Furthermore, the thermal conductivity of concrete is also strongly influenced by the w/c ratio [34] and cement content [35]. It has been established that lower aggregate thermal conductivity causes lower concrete thermal conductivity. This aspect could positively influence the usability of concrete with RMA due to its lower thermal conductivity caused by the low thermal conductivity of RMA, which ranges between 0.60 and 0.78 W/(m·K) [33]. The thermal conductivity of NA depends on its mineralogical characteristics, composition, and degree of crystallization. The heat conduction of NA with a crystalline structure is higher than amorphous and vitreous NA of the same composition [36].

In order to decrease the thermal conductivity of concrete, various kinds of thermal insulation materials have been added to self-insulating concrete. For instance, expanded polystyrene (EPS) beads, vermiculite, and glazed hollow beads have been used as insulating materials added to concrete. The utilization of thermal insulation materials as partial replacement of sand in concrete mixtures diminishes the mechanical properties, density, and thermal conductivity of concrete. Moreover, the decrease of these properties depends on the type of additional material [37,38,39,40,41,42,43,44,45,46]. The density of self-insulating concrete with recycled EPS ranges from 1070 to 1250 kg/m^3^, with the thermal conductivity between 0.34 and 0.5 W/(m.K) and compressive strength between 7.74 and 15.55 MPa at 28 days (see Table 1) [37]. In another study [38], two types of lightweight concrete were manufactured. One concrete mixture with a fresh density of 400 kg/m^3^ was produced with the strength of 3.0 MPa and thermal conductivity 0.09 W/(m.K), and another concrete mixture with a fresh density of 800 kg/m^3^ was produced with the strength of 13.0 MPa and thermal conductivity 0.25 W/(m.K). Polystyrene foamed concretes of densities ranging from 150 to 1200 kg/m^3^ with an EPS volume between 0% and 82% were compared with foamed concrete of 800 kg/m^3^ density without EPS [39]. The results of this study indicated a significant decline of compressive strength and a reduction of thermal conductivity caused by the increased EPS content. The concrete mixture containing 45% EPS had compressive strength of about 0.85 MPa and thermal conductivity of 0.16 W/(m·K), while the concrete mixture with 82% EPS had compressive strength of 0.08 MPa and thermal conductivity of 0.08 W/(m·K). Recycled EPS can be also used as a partial replacement of aggregate in self-insulating concrete for structural utilization [40].

Research on recycled materials as a partial or full replacement of NA in structural applications such as concrete blocks, paving blocks or floor blocks has already been published. The main reason for replacement of aggregate, which is the major component in concrete blocks, is the primary sources savings [50]. There are many recycled waste materials which is possible to use as partial or full replacement of aggregate in concrete blocks such as recycled concrete waste [51,52,53,54,55,56,57], crushed brick waste [11,58,59,60,61,62,63,64,65,66], glass waste [67,68,69,70], crump rubber waste [71,72,73], ceramic and tile industry waste [74], marble waste [75,76], plastic waste [77] and concrete slurry waste [78,79]. Moreover, due to its unique characteristics, the recycled materials could positively influence some properties of concrete blocks such as thermal conductivity, thermal resistance [65] or mechanical properties [67].

The use of RMA as a partial or full replacement of aggregate in structural concrete was examined for manufacturing precast prestressed beams [11], paving with precast concrete [58], and paving blocks or hollow tiles [59]. It was found that the most affected property of concrete was the modulus of elasticity, while compressive and tensile strengths were maintained at acceptable values for the full replacement of NA. The maximal acceptable replacement rate of RMA was found to be up to 35% for concrete with RMA in precast prestressed joists of building floors. Recycled aggregates from CDW containing more than 50% of waste concrete, more than 20% of waste clay bricks, and around 20% of cement or mortar stone were also used as a partial replacement of natural aggregates for preparing concrete masonry blocks suitable for indoor applications [60,61]. In this research, full blocks of 95% RA and hollow blocks of 75% RA were manufactured and tested.

This paper presents the environmentally based optimization of a concrete mixture containing recycled materials for mortarless masonry wall structures. Due to the low thermal conductivity of RMA and EPS, their utilization could have great potential for manufacturing concrete blocks for mortarless masonry walls of low-rise buildings, despite the decline of strength. From technical and/or economic viewpoints, the principle of mortarless masonry permits easy wall deconstruction for the most effective reuse of structural elements after their end of life.

## 2. Materials and Methods 

In total, 10 concrete mixtures were prepared and tested in order to verify the properties of concrete made using recycled masonry aggregate. One of them was a reference mixture with a natural aggregate only, and other mixtures contained recycled masonry aggregate and recycled expanded polystyrene in various ratios as a partial or full replacement of natural aggregate.

### 2.1. Recycled Aggregate

**Figure 1:**
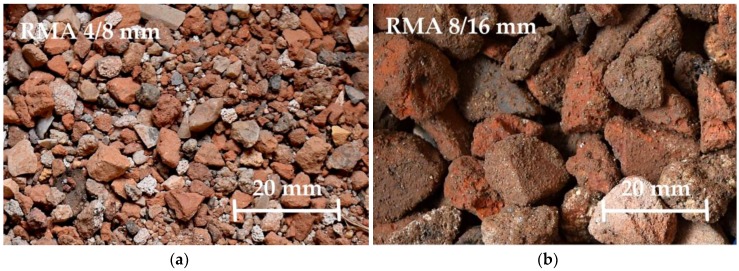
Recycled masonry aggregate. (**a**) RMA of fraction 4/8 mm; (**b**) RMA of fraction 8/16 mm

This research used one type of NA, two types of RMA, and one type of recycled EPS. Both types of RMA originated from construction and demolition waste and were delivered in fractions of 0–8 and 8–16 mm by a Czech recycling center (see Figure 1). For utilization as a substitute for fine-grained aggregate, in mixtures with an aggregate of fraction 0–16 mm in various replacement ratios, the fractions 0–4 and 4–8 mm were separated from the aggregate of fraction 0–8 mm in the laboratory. For mixtures containing a 0–8 mm fraction, RMA 0–8 mm was used without any laboratory treatment. Physical properties of RMA, especially water absorption, differ from NA. Therefore, the physical properties (see Table 2) are presented to show the differences in the materials used for the preparation of the concrete mixtures.

**Figure 2:**
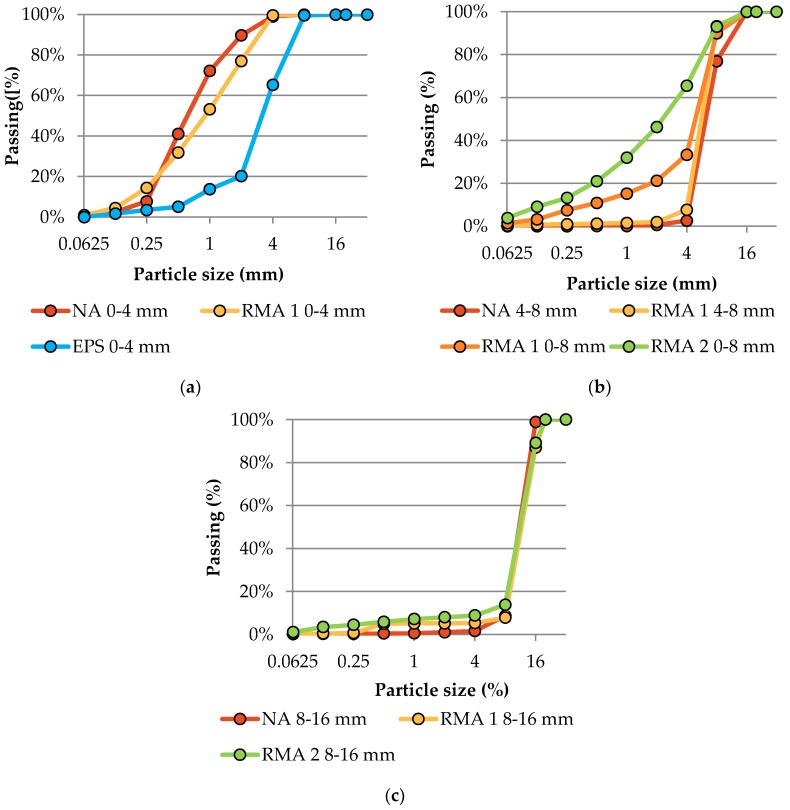
Sieving curves for natural aggregate, recycled masonry aggregate, and recycle expanded polystyrene used in concrete mixtures. (**a**) fraction 0–4 mm (**b**) fraction 4–8 and 0–8 mm (**c**) fraction 8–16 mm.

Selected properties of RMA were tested according to valid Czech standards. The properties most influencing the recipe design were tested. The basic physical properties of RMA are shown in Table 2, the granulometry is shown in graphs in Figure 2, and the composition of RMA is listed in Table 3.

### 2.2. Recycled Aggregate Concrete Mixtures

**Figure 3:**
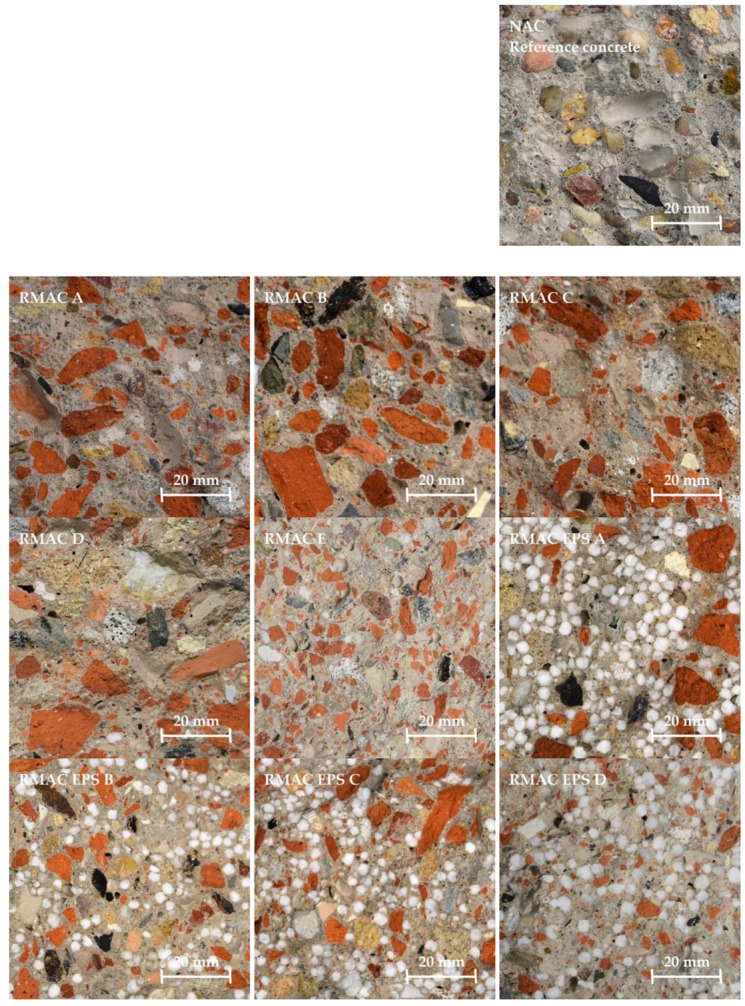
Types of recycled masonry aggregate concrete – tested samples.

Ten concrete mixtures with the same exposition class XF1, effective w/c ratio 0.5, and amount of cement CEM I 42.5 R 320 kg/m^3^ were prepared for laboratory measurements. One mixture of conventional concrete of strength class C30/37 only with NA of fraction 0–16 mm was manufactured as a reference to compare with the other mixtures in which NA was replaced in various ratios by RMA (five mixtures) and recycled EPS in addition (four mixtures). Two mixtures of RMA concrete (RMAC E and RMAC EPS D) only with RMA of fraction 0–8 mm was manufactured (see Table 4). The sample fragments of each material and the composition of RMA concrete mixtures are shown in Table 5 and Figure 3.

The physical, mechanical, deformation, and thermal properties were tested according to valid Czech standards. Samples of dimensions 100 × 100 × 400 mm, 150 × 150 × 150 mm, and 100 × 100 × 100 mm were used for testing.

### 2.3. Evaluation Methodology 

Samples were stored and cured in a stable laboratory environment during solidification and maturation, and after 28 days, the following properties were determined by laboratory tests: physical (density and capillary absorption), mechanical (compressive strength and tensile strength), deformation (static modulus of elasticity in compression), and thermal (volume heat capacity and thermal conductivity).

The mechanical properties, such as compressive strength, flexural strength, and static modulus of elasticity, were examined according to European and Czech standards. Water absorption capacity by immersion was tested on cubic specimens 100 × 100 × 100 mm. Specimens were treated by water, and after stabilization of weight, dried in an oven at a temperature of 105 ± 2 °C until stabilization of weight. The saturated surface-dried density and dry density were measured on these samples. Capillary water absorption was determined by measuring the rate of water absorption by capillaries. The ends of fractured prismatic specimens of 100 × 100 × approx. 150 mm, which were tested after the tensile strength test, were immersed in water up to a maximum height of 5 mm for 72 h or until their weight stabilized. The amount of water absorbed at different time intervals was measured by periodically weighing the surface-dried sample. Weighing intervals were 5 min, then 15 min for the first hour, then every hour for the first 6 h, and finally, every 12 h.

Measurement of thermal properties was done by the portable hand-held system ISOMET 2114 (Applied Precision Ltd., Bratislava, Slovakia) for measurement of the heat transfer properties of the materials. This applies a dynamic measurement method, which enables reducing the measurement time in comparison with steady-state measurement methods. It is equipped with a surface probe for measuring solid and hard materials. A flat surface of at least 60 mm diameter is satisfactory for the probe. Demand for the accuracy of the surface flatness increases as the thermal conductivity value of the tested material increases. The expected minimal thickness of the evaluated materials ranged from 20 to 40 mm depending on their diffusivity (conductivity).

Measured quantities and measurement ranges:

- Thermal conductivity λ (W/(m·K)): 0.04–6.00;
- Thermal diffusivity a (m^2^/s);
- Volume heat capacity c_ρ_ (J/(m^3^·K)): 4.0 × 10^4^ to 3.0 × 10^6^;
- Temperature T (°C): −15 to +50.

The thermal properties were tested on three cube samples of concrete of dimensions 100 × 100 × 100 mm for each mixture (see Table 3). The samples were tested under constant laboratory conditions. The temperature was 23 ± 3 °C.

## 3. Results and discussion

### 3.1. Physical Properties

**Figure 4:**
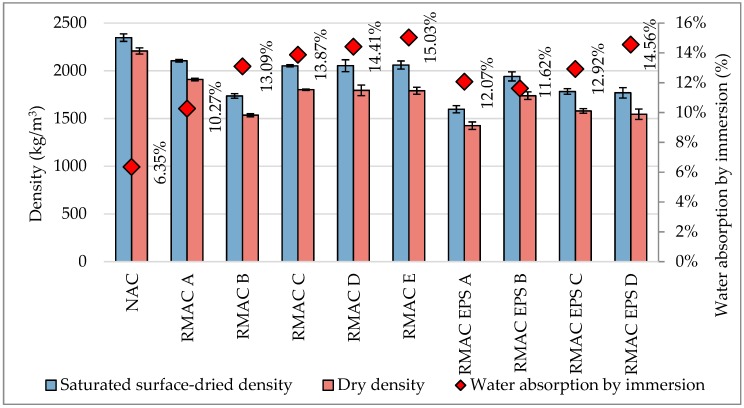
Comparison of water absorption by immersion of recycled aggregate concrete with RMA and EPS with conventional concrete.

**Figure 5:**
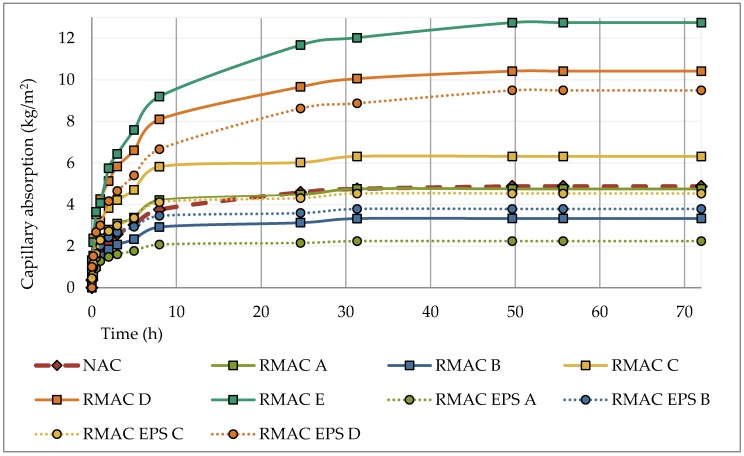
Comparison and progression of capillary water absorption of recycled aggregate concrete with RMA and EPS with conventional concrete.

The water absorption of buildings materials is important to know due to its influence on durability [80,81]. The values of density, water absorption by immersion, and capillary water absorption are shown in Table 6. Test results of the density showed lower values for all tested recycled aggregate concrete mixtures than the reference concrete. The density of recycled aggregate concrete declined with the increasing amount of RMA and EPS. The lowest value of density was measured for a mixture with 30% EPS, with a difference of 33%. The water absorption by immersion was approximately two times higher for the recycled aggregate concrete mixture than the reference concrete. Furthermore, the saturated surface-dried density, which was measured on saturated concrete samples, was the highest for reference mixture and the lowest for the mixture with the highest content of RMA and EPS (see Figure 4). In addition, the results of the capillary water absorption of concrete mixtures with 100% RMA showed similar results. On the contrary, capillary water absorption of concrete mixtures with EPS was mostly lower than that of the reference concrete (see Figure 5). The results of the density and water absorption by immersion confirm the results reported in previous studies [7,80,81,82,83].

### 3.2. Mechanical and Deformation Properties

**Figure 6:**
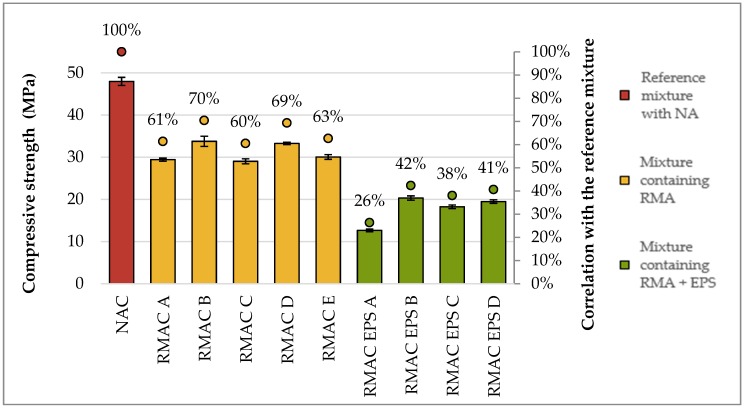
Comparison of compressive strength of recycled aggregate concrete with RMA and EPS with conventional concrete.

The compressive strength is the most important mechanical property of concrete blocks, which can be greatly enhanced by using recycled aggregate. The compressive strength of load-bearing wall blocks should not be less than 7 MPa [84]. The compressive strength and static modulus of elasticity were tested for all concrete mixtures because of their considerable importance for utilization as concrete blocks for wall structures. Further, flexural strength was also tested to better understand these materials. The values of compressive strength, flexural strength, and static modulus of elasticity are shown in Table 7. Test results of the compressive strength showed lower values for all tested RACs in comparison with the reference concrete. The compressive strength of RAC was influenced by the ratio of RMA and EPS. The highest compressive strength of RAC was measured for the concrete mixture with full replacement of aggregate by RMA. On the contrary, the lowest values of compressive strength were for mixtures with EPS (see Figure 6). As reported in previous studies [85,86,87], the static modulus of elasticity is a property that usually shows the greatest difference between natural aggregate concrete (NAC) and recycled aggregate concrete (RAC). The decline for full replacement rates is mostly more than 50%, which applied in this case too.

### 3.3. Thermal Properties

The lightweight and thermal insulation properties are the important functional characteristics of wall blocks for buildings [50]. The utilization of concrete blocks is not usually popular for wall structures of low-rise buildings due to its higher thermal conductivity, despite it being a material used for better heat stability in buildings. For this reason, it is very suitable to maintain heat capacity while increasing the thermal conductivity of the material. Previous studies found that the utilization of recycled materials such as recycled concrete aggregate [47], crumb rubber waste [72,88] or recycled EPS [40] leads to lower thermal conductivity of the recycled aggregate concrete. For this reason, this finding was also verified with recycled masonry aggregate concrete (RMAC) in this study. It was proved that the utilization of RMA for concrete wall masonry blocks leads to the lower thermal conductivity.

**Figure 7:**
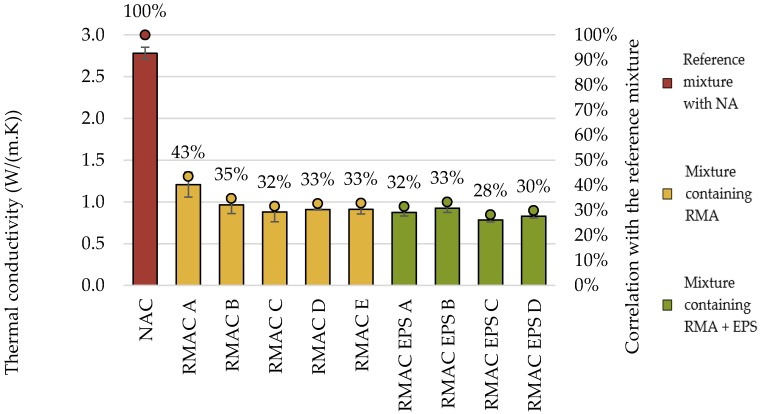
Comparison of thermal conductivity of recycled aggregate concrete with RMA and EPS with conventional concrete.

The thermal conductivity of concrete mixtures with RMA was approximately three times lower in comparison with NAC (see Figure 7). The thermal conductivity of concrete is influenced by the replacement rate and type of aggregate. However, the addition of EPS had no significant effect on this property. If 100% of the aggregate is substituted by RMA, the thermal conductivity will be similar to mixtures containing EPS. However, test results of the volume heat capacity showed similar values for all tested concrete mixtures, with the maximal difference up to 10% (see Table 8.). 

**Figure 8:**
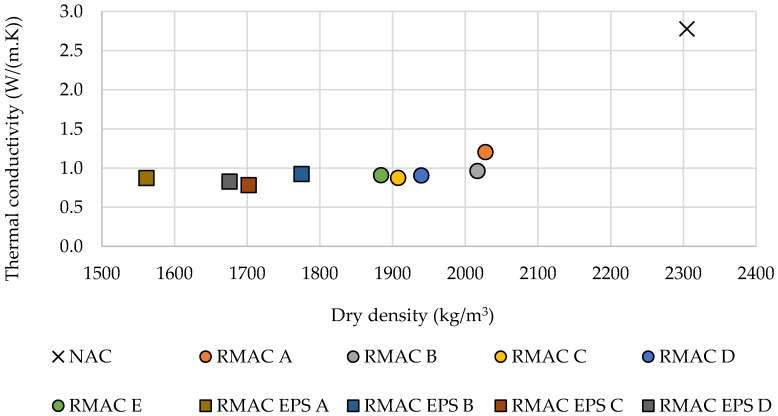
Effect of dry density on thermal conductivity for all tested concrete mixtures.

**Figure 9:**
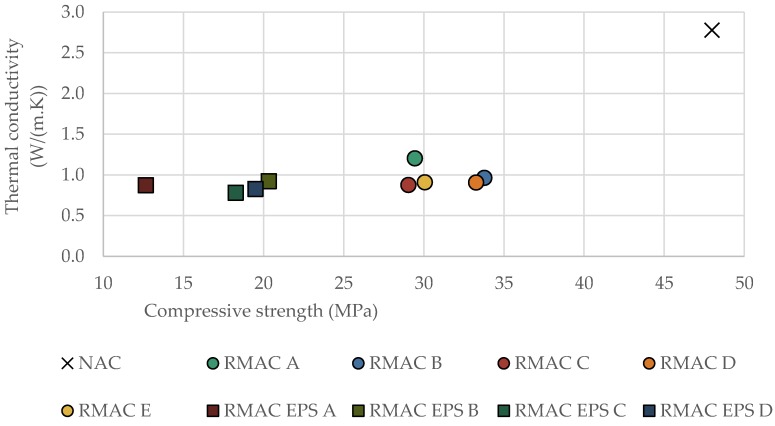
Effect of compressive strength on thermal conductivity for all tested concrete mixtures.

**Figure 10:**
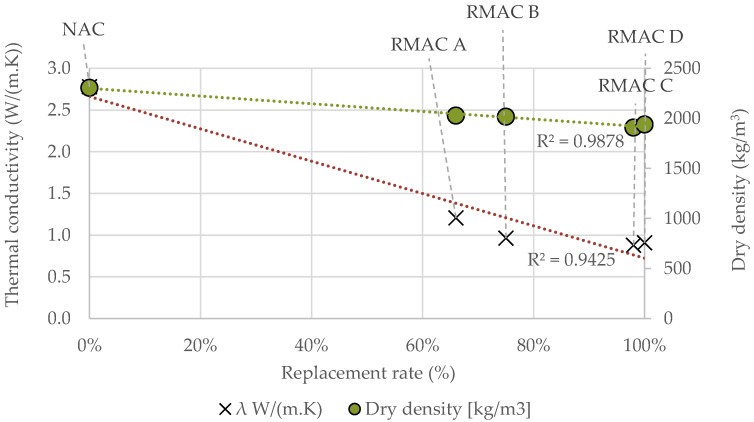
Effect of replacement rate on thermal conductivity and dry density.

It was found that the thermal conductivity of concrete slightly depends on its density and compressive strength (see Figure 8 and Figure 9). However, correlations were not as significant as expected. On the contrary, the results showed that the thermal conductivity and density were dependent on the replacement rate of the aggregate in the concrete mixture. The increasing amount of RMA in mixture led to a decrease in thermal conductivity and density (see Figure 10). In conclusion, the results showed that the best mixture in terms of thermal properties, density, and mechanical properties as well as recycled material content was RMAC D, which contained 100% fine and coarse RMA.

## 4. Conclusion

In this study, the environmental optimization and experimental verification of the physical, mechanical, and thermal properties of concrete containing various amounts of recycled masonry aggregate were examined and discussed. The final conclusions that have been reached can be summarized in the following points:

1. Recycled aggregate concrete has generally higher water absorption compared with conventional concrete. In this case, the water absorption by immersion and capillary water absorption were approximately two times higher. This aspect negatively influences the durability of concrete, especially freeze–thaw resistance. Nevertheless, it is not so important for insulated walls above ground level.
2. The use of recycled masonry aggregate as a partial or full replacement of natural aggregate in concrete mixtures negatively influences the mechanical properties of concrete, such as compressive strength, modulus of elasticity, and so forth. Here, the decline of compressive strength was between 30% and 75% and the decline of static modulus of elasticity was between 42% and 68% depending upon replacement rates.
3. The thermal conductivity of concrete with recycled masonry aggregate was approximately 70% lower than the value of the reference concrete with only a natural aggregate. The utilization of 30% recycled EPS for the concrete mixture with recycled masonry aggregate further decreased the thermal conductivity by about 5%. The volume heat capacity of concrete containing both types of recycled materials was similar to the volume heat capacity of concrete with natural aggregate.
4. The decrease of the thermal conductivity of recycled aggregate concrete depends on the replacement ratio of recycled masonry aggregate in concrete. This dependence applies to the same type and fraction of recycled aggregate.

As it was mentioned in previous studies about concrete blocks with recycled materials, the replacement of aggregate by recycled materials with their unique characteristics can positively influence the properties of concrete blocks for special applications [11,50,51,52,53,54,55,56,57,58,59,60,61,62,63,64,65,66,67,68,69,70,71,72,73,74,75,76,77,78,79]. The test results showed that the use of recycled aggregate as a replacement for natural aggregate in concrete positively influenced the thermal conductivity of concrete, although it negatively influenced the mechanical properties. On one hand, the better thermal conductivity of concrete masonry blocks from recycled aggregate concrete with RMA in comparison with conventional concrete masonry blocks reduces thermal insulation thickness while maintaining the same thermal properties of the structure. This leads to other material savings in addition to natural aggregate savings. On the other hand, maintaining the same mechanical properties might require the use of more cement. For these reasons, it is necessary to find the optimal way to use this type of recycled material. One possibility is using recycled aggregate concrete with recycled masonry aggregate to manufacture concrete blocks for mortarless masonry for low-rise buildings without high mechanical property and durability requirements. Finally, using mortarless masonry increases the potential for concrete masonry blocks to be reused.

## Figures and Tables

**Table 1:** Thermal properties of various materials in the dry state [33,36,37,47,48,49].

Type of Aggregate	Thermal Conductivity—λ	Type of Concrete	Thermal Conductivity—λ	Compressive Strength	Density
Aggregate	(W/(m·K))	Concrete	(W/(m·K))	(MPa)	(kg/m^3^)
NA-Basalt	4.03	Basalt concrete	2.26	N/A	N/A
NA-Limestone	3.15	Limestone concrete	2.03	N/A	N/A
NA-Siltstone	3.52	Siltstone concrete	2.21	N/A	N/A
NA-Quartzite	8.58	Quartzite concrete	2.77	N/A	N/A
RA-Concrete	2.22	RCAC 50%	0.90	34.7	2050
		RCAC 70%	0.80	39.1	2040
RA-Masonry	0.8	RMAC-various replacement	0.60–0.78	4.0	1480
RA-EPS	0.04	Concrete EPS content 55%	0.56	11.85	1140
		Concrete EPS content 65%	0.50	7.74	1070

RCAC: Recycled concrete aggregate concrete; RMAC: Recycled masonry aggregate concrete.

**Table 2:** Physical properties of particular fractions of used aggregates.

Types of Recycled Aggregate	Grading (mm)	Oven-Dried Particle Density (kg/m^3^)	Water Absorption Capacity (%)
Natural aggregate	0–4	2570	2
	4–8	2530	1.7
	8–16	2540	1.9
Recycled masonry aggregate 1	0–4	2200	16
	4–8	1970	13.8
	8–16	1950	11.9
Recycled masonry aggregate 2	0–8	1860	14.7
	8–16	2160	12.1
Recycled EPS	0–4	30	-

**Table 3:** Composition of recycled masonry aggregates A and B.

Material	Class	RMA 1		RMA 2	
		4–8 mm	8–16 mm	4–8 mm	8–16 mm
**Concrete (%)**	Rc	12.5%	30.9%	27%	25%
Unbound aggregates (%)	Ru	16.5%	13.7%	12%	29%
Ceramics (%)	Rb	67.9%	48.9%	61.0%	47.0%
Asphalt (%)	Ra	2.8%	2.8%	0%	0%
Lightweight particles (%)	FL	0.3%	0.0%	0%	0%
Natural soil and others (%)	X	0.0%	0.7%	27%	25%
Glass (%)	Rg	0.0%	2.9%	12%	29%

**Table 4:** Concrete mix proportion, per cubic meter.

Designation	NAC	RMAC A	RMAC B	RMAC C	RMAC D	RMAC E	RMAC EPS A	RMAC EPS B	RMAC EPS C	RMAC EPS D
Replacement ratio of aggregate (%)	0	66	75	98	100	95	76	76	98	83
Amount of EPS (kg)	0	0	0	0	0	0	6	4	4	6
Cement (kg)	320	320	320	320	320	320	320	320	320	320
Water (kg)	160	256	215	240	184	285	181	195	208	230
Sand (kg)	681	685	455	35	0	0	440	446	217	220
NCA 4/8 (kg)	541	0	0	0	0	96	0	0	0	88
NCA 8/16 (kg)	616	0	0	0	0	0	0	0	0	0
RFA 0/4 (kg)	0	0	0	459	529	0	0	0	250	0
RCA 0/8 (kg)	0	0	0	0	0	1267	0	0	0	712
RCA 4/8 (kg)	0	455	569	398	188	0	86	281	187	0
RCA 8/16 (kg)	0	719	493	534	772	0	562	535	557	0
w/c eff (–)	0.50	0.50	0.50	0.50	0.50	0.50	0.50	0.50	0.50	0.50
w/c (–)	0.50	0.81	0.68	0.75	0.58	0.89	0.56	0.60	0.65	0.72

**Table 5:** Composition of concrete mixtures.

RMAC Mixtures	Type of RMA	Coarse Fraction Repl.	Fine Fraction Repl.	EPS
RMAC A	RMA1	100%	0%	0%
RMAC B	RMA 1	100%	35%	0%
RMAC C	RMA 1	100%	95%	0%
RMAC D	RMA 2	100%	100%	0%
RMAC E	RMA 2	95%		0%
RMAC EPS A	RMA 2	100%	10%	30%
RMAC EPS B	RMA 1	100%	25%	18%
RMAC EPS C	RMA 1	100%	65%	18%
RMAC EPS D	RMA 2	85%		30%

**Table 6:** Average values of results of physical properties of concrete, including standard deviation.

Recycled Concrete Mixture		Density		Water Absorption by Immersion		Capillary Water Absorption
Designation		(kg/m^3^)	σ	(%)	σ	(kg/m^2^)
0	NAC	2305	9.13	6.35	2.81	4.87
1	RMAC A	2028	4.18	10.27	0.12	4.75
2	RMAC B	2017	11.87	13.09	0.36	3.33
3	RMAC C	1908	10.98	13.87	0.26	6.31
4	RMAC D	1939	4.67	14.41	0.08	10.41
5	RMAC E	1884	9.29	15.03	0.02	12.75
6	RMAC EPS A	1561	8.09	12.07	0.42	2.23
7	RMAC EPS B	1775	12.85	11.62	0.21	3.79
8	RMAC EPS C	1702	6.91	12.92	0.20	4.53
9	RMAC EPS D	1675	9.06	14.56	0.50	9.49

**Table 7:** Average values of results of mechanical properties of concrete, including standard deviation.

Recycled Concrete Mixture		Compressive Strength		Flexural Strength		Static Modulus of Elasticity	
Designation		(MPa)	σ	(MPa)	σ	(GPa)	σ
0	NAC	47.99	0.96	6.44	34.7	0.71	34.7
1	RMAC A	29.43	0.36	4.86	19.9	0.60	19.9
2	RMAC B	33.77	1.20	5.81	20.0	0.51	20.0
3	RMAC C	29.03	0.60	4.61	15.7	0.24	15.7
4	RMAC D	33.26	0.29	6.53	14.0	0.07	14.0
5	RMAC E	30.06	0.55	7.29	14.9	0.22	14.9
6	RMAC EPS A	12.65	0.33	3.22	11.0	0.17	11.0
7	RMAC EPS B	20.32	0.51	3.95	14.4	0.29	14.4
8	RMAC EPS C	18.26	0.43	3.26	12.4	0.70	12.4
9	RMAC EPS D	19.49	0.42	4.17	12.5	0.00	12.5

**Table 8:** Average values of results of thermal properties of concrete, including standard deviation.

Recycled Concrete Mixture		Thermal Conductivity—λ		Volume Heat Capacity—c_ρ_		Thermal Diffusivity—a	
Designation		(W/(m·K))	σ	*10^6^ (J/(m^3^·K))	σ	*10^−6^ (m^2^/s)	σ
0	NAC	2.778	0.15	1.773	0.15	1.573	0.09
1	RMAC A	1.207	0.12	1.790	0.02	0.676	0.07
2	RMAC B	0.965	0.00	1.691	0.04	0.571	0.01
3	RMAC C	0.879	0.06	1.654	0.01	0.531	0.04
4	RMAC D	0.908	0.04	1.666	0.05	0.545	0.02
5	RMAC E	0.911	0.05	1.626	0.03	0.561	0.03
6	RMAC EPS A	0.875	0.02	1.728	0.01	0.506	0.02
7	RMAC EPS B	0.925	0.02	1.792	0.03	0.516	0.01
8	RMAC EPS C	0.784	0.05	1.662	0.05	0.473	0.04
9	RMAC EPS D	0.830	0.04	1.617	0.05	0.513	0.02
